# Avian Influenza Virus PB1 Gene in H3N2 Viruses Evolved in Humans To Reduce Interferon Inhibition by Skewing Codon Usage toward Interferon-Altered tRNA Pools

**DOI:** 10.1128/mBio.01222-18

**Published:** 2018-07-03

**Authors:** Bartram L. Smith, Guifang Chen, Claus O. Wilke, Robert M. Krug

**Affiliations:** a Department of Molecular Biosciences, John Ring LaMontagne Center for Infectious Disease, Institute for Cellular and Molecular Biology, the University of Texas at Austin, Austin, Texas, USA; b Department of Integrative Biology, Center for Computational Biology and Bioinformatics, Institute for Cellular and Molecular Biology, the University of Texas at Austin, Austin, Texas, USA; National Institute of Allergy and Infectious Diseases

**Keywords:** codon usage, evolution, influenza A virus, influenza PB1 protein, interferon

## Abstract

Influenza A viruses cause an annual contagious respiratory disease in humans and are responsible for periodic high-mortality human pandemics. Pandemic influenza A viruses usually result from the reassortment of gene segments between human and avian influenza viruses. These avian influenza virus gene segments need to adapt to humans. Here we focus on the human adaptation of the synonymous codons of the avian influenza virus PB1 gene of the 1968 H3N2 pandemic virus. We generated recombinant H3N2 viruses differing only in codon usage of PB1 mRNA and demonstrated that codon usage of the PB1 mRNA of recent H3N2 virus isolates enhances replication in interferon (IFN)-treated human cells without affecting replication in untreated cells, thereby partially alleviating the interferon-induced antiviral state. High-throughput sequencing of tRNA pools explains the reduced inhibition of replication by interferon: the levels of some tRNAs differ between interferon-treated and untreated human cells, and evolution of the codon usage of H3N2 PB1 mRNA is skewed toward interferon-altered human tRNA pools. Consequently, the avian influenza virus-derived PB1 mRNAs of modern H3N2 viruses have acquired codon usages that better reflect tRNA availabilities in IFN-treated cells. Our results indicate that the change in tRNA availabilities resulting from interferon treatment is a previously unknown aspect of the antiviral action of interferon, which has been partially overcome by human-adapted H3N2 viruses.

## IMPORTANCE

Pandemic influenza A viruses that cause high human mortality usually result from reassortment of gene segments between human and avian influenza viruses. These avian influenza virus gene segments need to adapt to humans. Here we focus on the human adaptation of the avian influenza virus PB1 gene that was incorporated into the 1968 H3N2 pandemic virus. We demonstrate that the coding sequence of the PB1 mRNA of modern H3N2 viruses enhances replication in human cells in which interferon has activated a potent antiviral state. Reduced interferon inhibition results from evolution of PB1 mRNA codons skewed toward the pools of tRNAs in interferon-treated human cells, which, as shown here, differ significantly from the tRNA pools in untreated human cells. Consequently, avian influenza virus-derived PB1 mRNAs of modern H3N2 viruses have acquired codon usages that better reflect tRNA availabilities in IFN-treated cells and are translated more efficiently.

## INTRODUCTION

Many studies have shown that adaptation of avian influenza A virus segments to humans requires mutations in nonsynonymous codons to change specific amino acids in one or more viral proteins (1). In contrast, the role of synonymous mutations in the adaptation to humans is not known. Codon usage biases, which differ among species (2–7), are likely caused by selection for translational efficiency (8–10) and accuracy (11–13), largely mediated by the availability of isoaccepting tRNAs (14). Because influenza A virus depends on the host translational machinery, the codon usage of avian influenza virus genes would be expected to change as the virus adapts to a human host. Surprisingly, changes in codon usage of these avian genes have been reported to move away from the presumed optimal codon usage in humans over time, an observation that has not been explained (15).

Here we focus on the adaptation in humans of the codons of the avian influenza virus PB1 gene that was incorporated into the 1968 H3N2 pandemic virus (16). The PB1 protein encoded by this gene is one of the subunits of the viral polymerase (17–19). The PB1 gene of H3N2 viruses has a history different from those of the other seven gene segments in these viruses. Six of the other gene segments in H3N2 viruses (encoding the PA and PB2 polymerase subunits, the neuraminidase, the nucleoprotein, the M1/M2 proteins, and the NS1/NS2 proteins) were incorporated into human influenza A viruses prior to 1968. The avian influenza virus neuraminidase gene segment was incorporated into a pandemic human virus in 1957 (16), but it is not known with certainty when the other five gene segments were derived from avian influenza viruses. Although the hemagglutinin (HA) gene segment, like the PB1 gene segment, was introduced into human viruses in 1968, the HA gene has undergone extensive divergence at the amino acid level since its introduction. This nonsynonymous divergence would be expected to obscure synonymous codon changes.

Here we identify the viral phenotype resulting from the PB1 codon changes that evolved since the introduction of the avian influenza virus PB1 gene into the 1968 pandemic H3N2 virus. Specifically, we demonstrate that the evolved codon usage of PB1 mRNA enhances the replication of modern H3N2 viruses in interferon (IFN)-treated human cells without affecting virus replication in untreated cells, thereby partially alleviating the antiviral activities induced by IFN. We focus on how the changed codons lead to reduced inhibition of virus replication by IFN and demonstrate that this reduced inhibition is explained by the evolution of PB1 codons skewed toward the tRNA pools in IFN-treated human cells which, as shown here, differ significantly from the tRNA pools in untreated human cells. Consequently, avian influenza virus-derived PB1 mRNAs of modern H3N2 viruses acquire codon usages that better reflect tRNA availabilities in IFN-treated cells and are likely translated more efficiently. Our results indicate that the change in tRNA availabilities resulting from IFN treatment is a previously unknown aspect of the antiviral action of IFN which has been partially overcome by human-adapted H3N2 viruses.

## RESULTS

### Evolution of codon usage of the avian influenza virus-derived H3N2 PB1 segment relative to human codon usage.

We used the codon adaptation index (CAI) (20) to compare the codon usage of the PB1 mRNA of influenza A virus isolates to human codon usage. More than 10,000 human PB1 mRNA sequences from three influenza A virus lineages (H1N1, H2N2, and H3N2) and influenza B virus lineages isolated between 1932 and 2015 were downloaded from the GISAID EpiFlu influenza sequence database (http://gisaid.org). We chose to focus on the PB1 gene because it is the only avian influenza virus gene encoding an internal virion protein that has been introduced several times into human influenza A viruses. The CAI of PB1 mRNAs for the three influenza A virus lineages shows an overall decline over time (see Fig. S1a in the supplemental material), verifying previous findings (15), whereas the PB1 mRNA of influenza B viruses did not show this decline over time (Fig. S1a). In addition, this decline in CAI did not occur in the PB1 mRNAs of avian influenza viruses during this time period (Fig. S1b). Because H2N2 viruses did not circulate very long in the human population and because there are few early (pre-1940) sequences for H1N1 viruses, we focused on the PB1 gene segment of H3N2 viruses that have been circulating in the human population since 1968. This analysis benefits from a wealth of H3N2 PB1 sequence data.

10.1128/mBio.01222-18.2 FIG S1  CAI values over time since 1968 for (a) avian influenza virus, influenza B virus, H1N1, and H2N2 PB1 genes and (b) each segment of H3N2 viruses. Download FIG S1, PDF file, 0.2 MB. Copyright © 2018 Smith et al. 2018 Smith et al. This content is distributed under the terms of the Creative Commons Attribution 4.0 International license.

**FIG 1:**
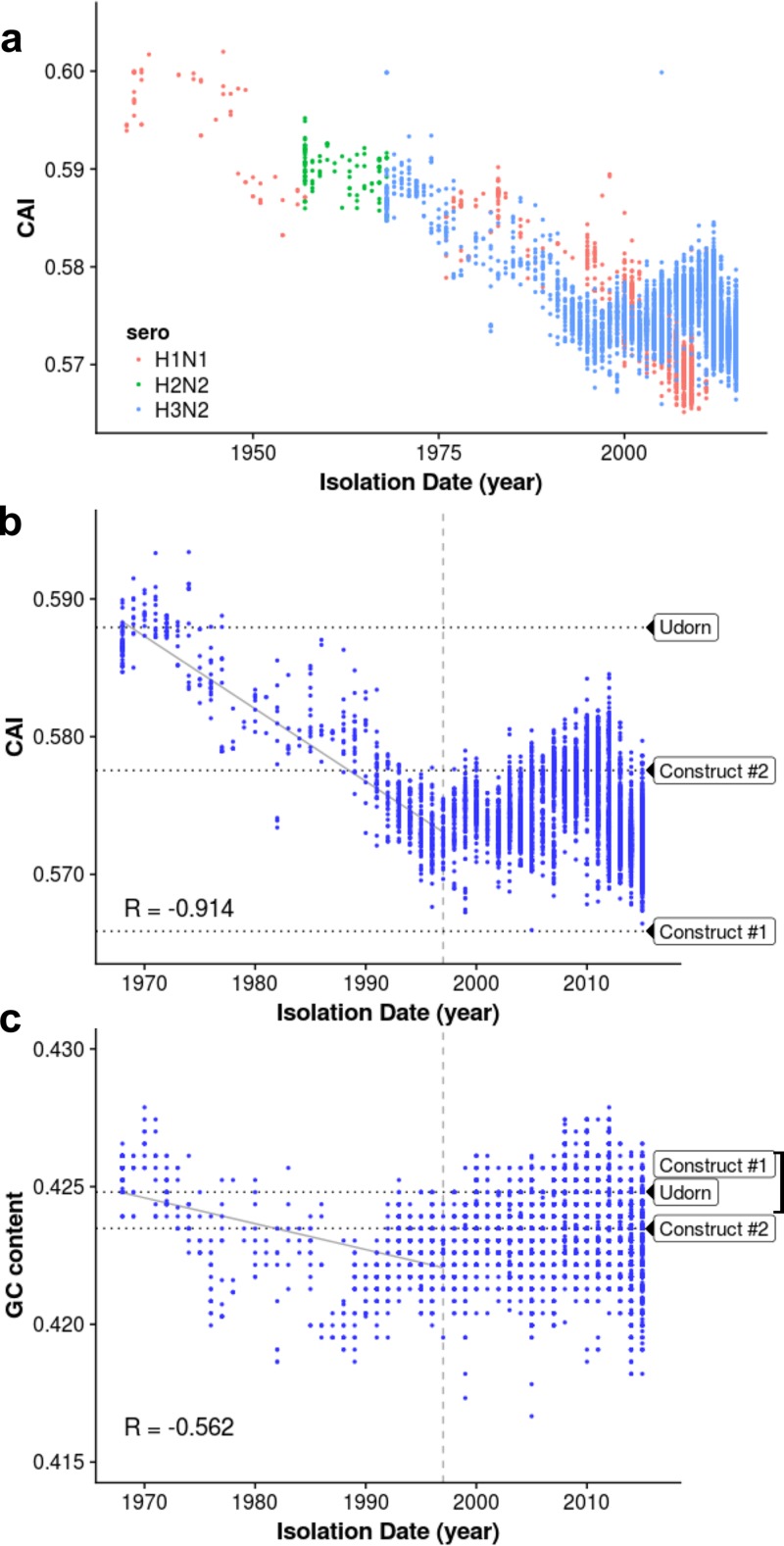
The CAI values of PB1 mRNAs of H1N1, H2N2, and H3N2 influenza A viruses show a decline over time after the introduction of the avian influenza virus PB1 segment into a human-infecting virus in 1968. (a) CAI values of PB1 mRNAs of H1N1, H2N2, and H3N2 influenza A viruses and of influenza B virus plotted against year of isolation since 1968. (b) CAI values of PB1 mRNAs of H3N2 viruses plotted against year of isolation. The CAI values of WT Ud virus and the two codon-altered Ud viruses are shown. The horizontal dotted lines represent the linear regression across the first 30 years of circulation, and the 30-year mark is denoted by the dashed vertical line. The *P* value of the linear regression, determined by the reshuffling test described in the text, is 0.017. (c) GC content of PB1 mRNAs of H3N2 viruses plotted against circulation time. The GC content of WT Ud virus and the two codon-altered Ud viruses is shown. The horizontal dotted lines represent the linear regression across the first 30 years of circulation, and the 30-year mark is denoted by the dashed vertical line. The *P* value of the linear regression, determined by the reshuffling test, is 0.509. See Fig. S1.

As shown in Fig. 1a and b, the CAI of the PB1 mRNAs of H3N2 viruses shows a strong decline for approximately 30 years after 1968. The CAI then levels out, indicating that the PB1 mRNAs of the more recent H3N2 isolates likely achieved a codon usage bias that balances the selective and mutational pressures on the synonymous codon usage of the PB1 gene. To demonstrate that the resulting trend in CAI values is significant and was not caused by the phylogenetic relationship between influenza A virus isolates, a reshuffling test was performed (see Text S1 in the supplemental material). The CAI values of other H3N2 gene segments do not show clear-cut trends over time (Fig. S1b). Because GC content can be a determinant of codon usage bias (21), we also plotted the GC content of the PB1 mRNAs of each H3N2 virus isolate against circulation time (Fig. 1c). A significant trend was not evident, and the PB1 mRNAs of early H3N2 viruses exhibit GC content that is still present in the PB1 mRNAs of the more recent H3N2 virus isolates, indicating that GC content does not drive the changes in the observed CAI values.

10.1128/mBio.01222-18.1 TEXT S1  Supplemental Materials and Methods. Download TEXT S1, PDF file, 0.4 MB. Copyright © 2018 Smith et al. 2018 Smith et al. This content is distributed under the terms of the Creative Commons Attribution 4.0 International license.

### Codon usage of recent H3N2 virus isolates enhances replication in IFN-treated human cells but not in untreated human cells.

To determine whether the codon changes in PB1 mRNA observed over time affect virus replication, we generated two different recombinant viruses whose PB1 CAI values reflect the CAI values of recent H3N2 viruses. The 1972 H3N2 Udorn/72 (Ud) isolate is an appropriate model for the codon usage (CAI) of early virus isolates because it was isolated only 4 years after the 1968 recombination event that created the H3N2 lineage and encodes a PB1 mRNA with a CAI similar to that of other early H3N2 viruses. We used the Ud virus gene segments as a backbone to generate influenza A viruses that express PB1 mRNAs with codon usages that give the gene a CAI score similar to that of modern H3N2 viruses. Because these codon-altered PB1 mRNAs encode the amino acid sequence of the Ud wild-type (WT) PB1 protein, we are comparing viruses that differ only between modern and original synonymous codon biases in PB1 mRNA without any potentially confounding nonsynonymous changes in PB1 or any other viral segment that might have developed from 1972 to the present. To prevent potentially confounding effects from the existence of a frameshifted alternate reading frame, we eliminated the synthesis of the small PB1-F2 protein by mutating its start codon in the WT PB1 and the two PB1 constructs. Deletion of PB1-F2 should not affect our results because previous studies showed that the elimination of the PB1-F2 protein does not affect influenza A virus replication in tissue culture cells, including human respiratory cells (22, 23). Also, we did not introduce base changes in the PB1 packaging sequences of the two PB1 constructs, specifically, the 84 bases and 103 bases at the 3′ and 5′ ends of PB1, respectively, and the bases in the 290-to-303 region of PB1 (24–26).

We made two codon-altered constructs of Ud PB1 by introducing randomized synonymous codon changes using two different methods. Construct 1 was made using a program that introduced random synonymous changes into the sequence of PB1 that decreased the CAI value of its mRNA while keeping the GC content constant. This was done iteratively until a sequence was obtained with a CAI value of approximately 0.560. Construct 2 was designed by generating a table of RSCU (relative synonymous codon usage) data for two dozen randomly chosen H3N2 PB1 sequences isolated in 2014. The number and type of synonymous codon changes needed to convert the RSCU of Ud PB1 to the average RSCU of the 2014 PB1 were determined. These changes were distributed randomly across the PB1 sequence. Construct 1 contains changes in 43 synonymous codons, and construct 2 has changes in 50 synonymous codons. The sequences of these two constructs are shown in the accompanying Zenodo citation. Importantly, the changes in the synonymous codons of the two constructs were almost entirely distinct, with an overlap of only three synonymous codon changes.

**FIG 2:**
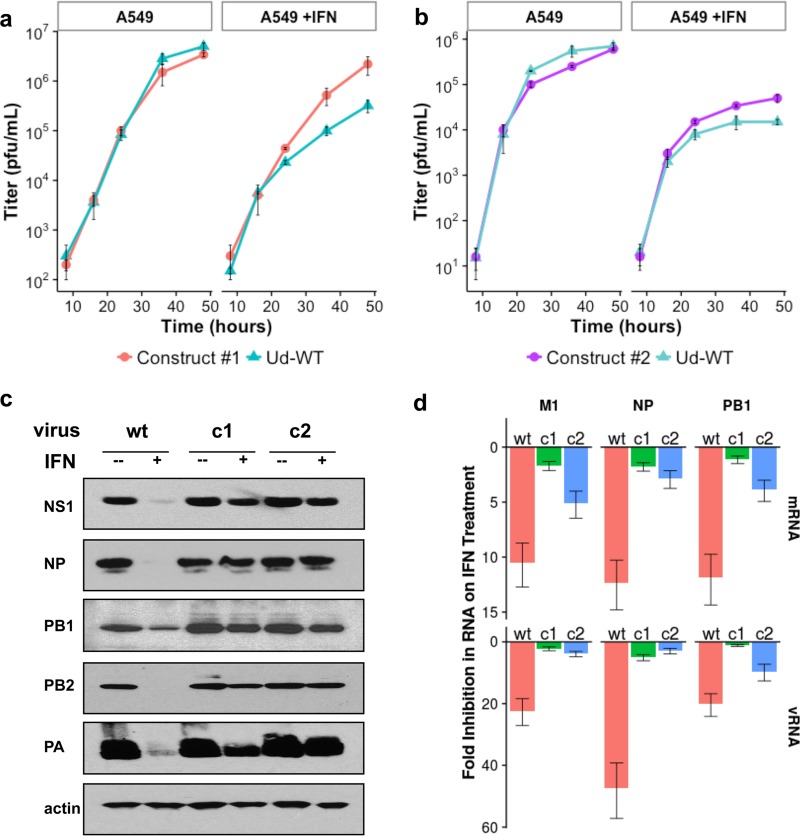
Recombinant Ud viruses expressing a PB1 mRNA with CAI values similar to those of recent H3N2 viruses replicate better than the WT 1972 Ud virus in IFN-treated human cells. Cells were pretreated with or without 1,000 IU/ml IFN for 36 h. (a) Growth curves, carried out in triplicate, of virus construct 1 and WT Ud viruses in A549 cells infected at a MOI of 0.01. The means are reported here, with error bars indicating standard errors. (b) Growth curve of construct 2 and WT Ud viruses in A549 cells infected at a MOI of 0.01. (c) Viral protein production in A549 cells infected with a MOI of 5 PFU/cell of construct 1, construct 2, or WT Ud virus. At 9 h after infection, cell extracts were immunoblotted with appropriate antibodies to detect the indicated viral proteins or actin (see Text S1). (d) RT-PCR results for the mRNAs and vRNAs encoded by the (M1) NP and PB1 genes. RNA was isolated from IFN-treated and mock treatment cells at 3 h postinfection of 5 PFU/cell of construct 1, construct 2, or WT Ud virus, and the fold inhibition caused by IFN treatment was measured in triplicate by the threshold cycle (−ΔΔ*C*_*T*_) method. Both constructs showed significantly reduced inhibition of viral RNA synthesis due to IFN treatment compared to the WT (*P* < 0.05 [two-tailed *t* test]) for all measured RNAs. Error bars (standard errors) are shown.

As shown above, changes in only approximately 5% of the PB1 mRNA codons were sufficient to transform the 1972 Ud CAI to the lower CAI of modern H3N2 viruses. Although it seemed unlikely that a reduced CAI value for PB1 mRNA would enhance virus replication, we nevertheless tested this possibility. Accordingly, human A549 lung epithelial cells were infected with WT Ud virus, construct 1 virus, or construct 2 virus at a low multiplicity of infection (MOI) (number of plaque-forming units/cell), resulting in multiple rounds of replication (Fig. 2a and b). Little or no difference in the rate or extent of virus replication was observed in comparisons between the WT Ud virus and the two viruses expressing codon-altered PB1 mRNAs. Hence, changes in the translation of the two codon-altered PB1 mRNAs in infected human cells were not detected, indicating that the codon changes in PB1 mRNA over time do not enhance influenza A virus replication in human cells.

It has been shown that the amount of tRNAs available in a cell, i.e., tRNA pools, can change under different conditions (27, 28). In addition, it has been reported that viruses can adapt to take advantage of these differential tRNA availabilities to control the expression level of viral proteins (29). As a substantial part of the infection cycle of influenza virus in humans takes place while the host is mounting a strong antiviral response, we decided to test the codon-altered constructs in human cells in which the antiviral state was induced by interferon (IFN) treatment, resulting in enhanced or decreased expression of hundreds of cellular genes (30).

To determine whether the synonymous changes introduced into the PB1 mRNAs of the two virus constructs improved replication in cells in the IFN-induced antiviral state, A549 cells were pretreated with 1,000 U/ml IFN, followed by infection with WT Ud virus or with one of the virus constructs at a low MOI. As shown in Fig. 2a and b, the two construct viruses grew to levels approximately 10-fold higher than those seen with the WT Ud virus in IFN-treated cells. To assay the effect of the codon changes on the synthesis of viral proteins and viral RNAs (vRNAs), A549 cells were infected with a high MOI of either the WT Ud or one of the two construct viruses. In the absence of IFN pretreatment, no significant difference in the amount of five viral proteins at 9 h after infection was detected between cells infected with WT Ud virus and the two construct viruses, demonstrating that the codon changes in PB1 mRNA over time do not affect high-multiplicity infections in untreated human cells. However, when A549 cells were pretreated with IFN, production of the five viral proteins that were measured, namely, the NS1 protein, the nucleoprotein (NP), and the three protein subunits of the viral polymerase (PB1, PB2, and PA), was severely reduced in WT Ud virus-infected cells, which was not the case in cells infected with the construct viruses (Fig. 2c), indicating that enhanced replication of the two construct viruses also occurred in high-MOI infections of IFN-treated A549 cells.

To determine whether the increased levels of viral protein production are attributable to increased levels of the viral mRNAs that were produced, we used quantitative real time-PCR (RT-PCR) to measure the levels of three viral mRNAs, encoding the M1 (matrix protein), NP, and PB1 proteins (Fig. 2d). The levels of all three viral mRNAs were reduced by IFN treatment to a much lower extent in construct 1 and construct 2 virus-infected cells than in WT Ud virus-infected cells, indicating that the higher levels of viral proteins in IFN-treated construct virus-infected cells were due to increased synthesis of viral mRNAs. In addition, quantitative RT-PCR analysis showed that the corresponding negative-sense viral RNAs (vRNAs) were reduced to a much lower extent in IFN-treated construct virus-infected cells than in WT Ud virus-infected cells, indicating that overall viral RNA synthesis was less reduced in IFN-treated, construct virus-infected cells and hence that the levels of viral polymerase activity in construct virus-infected cells are less attenuated by IFN treatment than the levels in WT Ud virus-infected cells.

It has been reported that the presence of CpG dinucleotides or CpG dinucleotides in an A/U context in an influenza viral mRNA affects the host antiviral response (31). Our constructs introduced fewer CpG changes than have been tested in previous studies; also, the numbers of changes compared to the WT virus differed significantly between the two constructs. The codon changes introduced into construct 1 increased the number of CpG dinucleotides (+11) and CpG dinucleotides in an A/U context (+2) in PB1 mRNA compared to WT Ud PB1 mRNA. Such increases were not introduced into construct 2; the number of CpG dinucleotides in PB1mRNA decreased (−4), and the number of CpG dinucleotides in an A/U context was unchanged. Because the two virus constructs exhibited the same phenotype in IFN-treated and untreated human cells, it is unlikely that this phenotype was due to the content of CpG dinucleotides or CpG dinucleotides in an A/U context.

### The tRNA pool in IFN-treated human cells differs from the tRNA pool in untreated cells.

**FIG 3:**
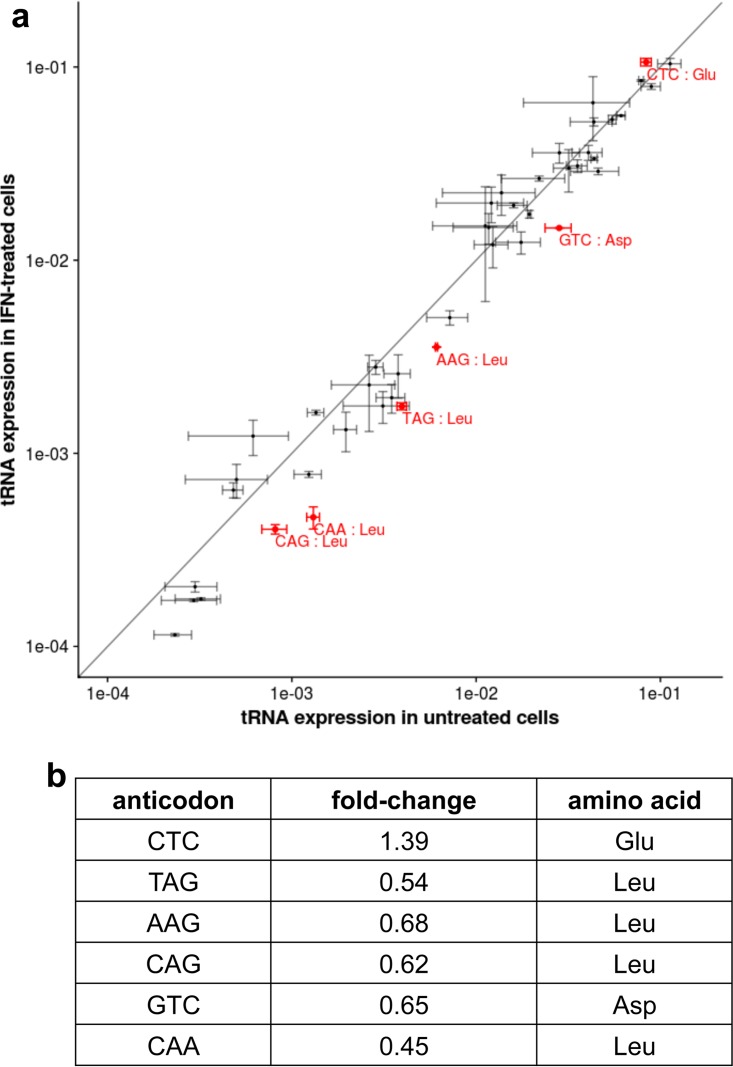
Levels of different tRNAs in IFN-treated human A549 cells compared to the tRNA levels in untreated cells. (a) Mean of the log of the proportion of total tRNAs of each tRNA anticodon in untreated cells versus the log proportion of total tRNAs in IFN-treated cells across three replicates. Error bars indicate standard errors. Six anticodons (marked in red) showed a statistically significant difference between untreated and IFN-treated cells (*P* [adjusted] < 0.05) using DESeq2. The solid line shows the values corresponding to tRNA anticodons that were present in equal amounts in IFN-treated and untreated cells. (b) Fold change in the six tRNA anticodons that differed significantly (adjusted *P* < 0.05) between IFN-treated and untreated cells, shown with their encoded amino acid.

The virus replication results described above show that the synonymous codon usage of PB1 mRNAs has evolved over time to take advantage of changes in human cells induced by IFN treatment. This codon evolution could represent an adaptation to IFN-induced alteration of cellular tRNA pools. To test this hypothesis, we first compared the tRNA pools in IFN-treated and untreated A549 human cells by high-throughput sequencing of tRNAs using a thermostable group II intron reverse transcriptase (TGIRT) that copies structured tRNAs with high fidelity (32). We obtained more than 1 × 10^8^ sequencing reads which aligned with tRNA genes in the human genome in all replicates from both IFN-treated and untreated cells. The reads that aligned with each tRNA gene were grouped by anticodon. We plotted the amounts of each tRNA anticodon as a proportion of all tRNAs in untreated cells versus the amounts of the tRNA anticodon as a proportion of all tRNAs in IFN-treated cells (Fig. 3a). The solid line in this graph shows the values of tRNA anticodons that were present in equal amounts in IFN-treated and untreated cells. The value for the determined amount of each anticodon is shown with bars denoting the standard errors based on three replicate determinations. Statistical analysis was carried out using differential expression analysis in the DESeq2 program, yielding an adjusted *P* value for each anticodon (33).

The amounts of six tRNA anticodons (denoted in red) differed significantly (adjusted *P* < 0.05) between IFN-treated and untreated cells. In Fig. 3b, these six tRNA anticodons are grouped with their encoded amino acids. Remarkably, four of the six tRNA anticodons whose levels decreased to different degrees in IFN-treated cells encode Leu. These results demonstrate that there are significant differences in the tRNA pools between IFN-treated and untreated A549 human cells.

### PB1 mRNA evolved its codon usage to adapt to a certain extent to the tRNA pools in IFN-treated human cells.

To identify how changes in tRNA availability affect the evolution of synonymous codon usage in the PB1 gene, and because the observed changes in usage of any single given codon are quite small, we developed a new metric denoted as the relative tRNA adaptation index (rtAI) to provide a single cumulative value to describe the change in codon usage of a gene related to changing tRNA availabilities. This metric compares the levels of availability of isoaccepting tRNAs in two sequenced tRNA pools, i.e., in the present study, the tRNA pools in IFN-treated cells versus the tRNA pools in untreated cells, for all the codons in an mRNA of interest, namely, PB1 mRNA in the present study. The specifics of these calculations are described in Text S1. A higher value of rtAI indicates that the codon usage of a PB1 mRNA is preferred in IFN-treated cells compared to untreated cells with respect to the availability of isoaccepting tRNAs in these two states of human cells. It should be emphasized that the rtAI value does not predict the overall synthesis of the PB1 protein or the overall replicative fitness of the virus but rather only how we expect the PB1 mRNA to be translated relatively between IFN-treated and untreated human cells.

**FIG 4:**
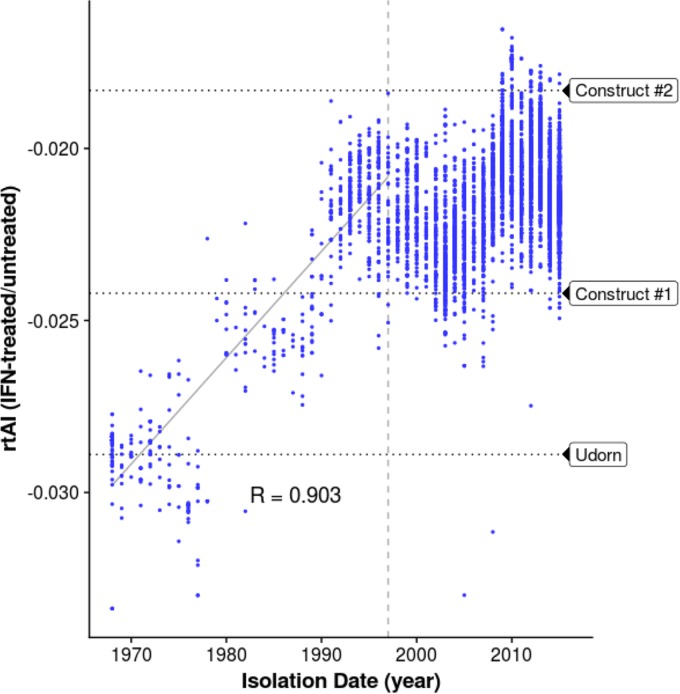
The rtAI values of H3N2 PB1 mRNAs for IFN-treated human cells versus untreated cells increased over time after 1968. The rtAI values for the two codon-altered virus constructs and WT Ud virus are shown. The horizontal dotted lines represent the linear regression across the first 30 years of circulation, and the 30-year mark is denoted by the dashed vertical line. The *P* value of the linear regression, determined by the reshuffling test, is 0.046. See Fig. S2.

As shown in Fig. 4, the rtAI values of the mRNAs encoded by H3N2 PB1 genes increased over time for approximately 30 years after the introduction of the avian influenza virus-derived PB1 gene into human viruses in 1968, demonstrating that during this period of time the avian influenza virus-derived PB1 coding sequence was evolving a synonymous codon usage in its mRNA which was skewed toward isoaccepting tRNAs in IFN-treated cells. After approximately 30 years, the rtAI values leveled off, indicating that the PB1 mRNAs encoded by the PB1 gene of the more recent H3N2 isolates have achieved an equilibrium point with respect to the relative levels of availability of isoaccepting tRNAs of IFN-treated versus untreated human cells. Virus constructs 1 and 2 exhibited the expected phenotype caused by adaptation to the differential tRNA availabilities that were measured, namely, less inhibition of replication than WT Ud virus resulting from IFN treatment (Fig. 2a and b). Of the codons that were changed to generate these two virus constructs, 13 and 10 codons for construct 1 and construct 2, respectively, were identified in Fig. 3 as having isoaccepting tRNAs with statistically significant differential availabilities in IFN-treated versus untreated cells. Because the rtAI values of these two construct viruses are at the upper and lower borders of the rtAI values of modern H3N2 viruses, it is likely that all modern H3N2 viruses share the phenotype of these two virus constructs, namely, reduced inhibition by IFN. In contrast to the PB1 gene, the rtAI values of other virus gene segments do not show clear-cut trends over time (Fig. S2).

10.1128/mBio.01222-18.3 FIG S2  rtAI values over time since 1968 for (a) avian influenza virus, influenza B virus, H1N1, and H2N2 PB1 genes and (b) each segment of H3N2 viruses. Download FIG S2, PDF file, 0.2 MB. Copyright © 2018 Smith et al. 2018 Smith et al. This content is distributed under the terms of the Creative Commons Attribution 4.0 International license.

To determine whether the observed differences in tRNA abundance between IFN-treated and untreated cells have influenced codon usage of human mRNAs, we calculated the rtAIs of human mRNAs, including both constitutively expressed mRNAs and mRNAs of IFN-regulated genes (IRG), which can be increased or decreased by IFN (30). For this purpose, we used the published results of a microarray expression experiment in A549 cells whose data are available on EMBL-EBI (accession no. E-GEOD-5542) (34). We compared the log_2_-fold change in the amount of each human mRNA caused by IFN treatment to the rtAI of each mRNA (Fig. S3a). This analysis revealed the surprising result that IFN-regulated host mRNAs (IRG mRNAs), compared to non-IRG host mRNAs, are restricted to rtAI values close to zero, indicating that these mRNAs are likely translated similarly in untreated and IFN-treated cells. To determine whether the rtAI scores close to zero are caused by the usage of codons differing in isoaccepting tRNA availability that balance out or are caused by the specific avoidance of codons recognized by isoaccepting tRNAs that change in availability upon IFN treatment, we developed a version of rtAI referred to as absolute rtAI. This metric, unlike rtAI, measures the overall change in isoaccepting tRNA availability for the codons of a given mRNA, regardless of whether the levels of these tRNAs increase or decrease upon IFN treatment. The specifics of these calculations are described in Text S1. A higher value of absolute rtAI means that the codons used by the mRNA are recognized by isoaccepting tRNAs that change in availability as a consequence of IFN treatment, and a lower value means that the codon usage of the mRNA avoids isoaccepting tRNAs that change in availability as a consequence of IFN treatment. As shown in Fig. S3b, IRG mRNAs are restricted to an absolute rtAI score close to zero compared to non-IRG mRNAs, indicating that IRG mRNAs specifically avoid codons recognized by isoaccepting tRNAs that change in availability between IFN-treated and untreated cells. These results suggest that the change in tRNA availabilities resulting from IFN treatment is predominately an antiviral response and that human IRG mRNAs avoid the use of codons that are recognized by the tRNAs that change greatly in abundance upon IFN treatment.

10.1128/mBio.01222-18.4 FIG S3  (a) Comparison of the log_2_ change in the amount of each human mRNA (including both IRG and non-IRG mRNAs) caused by IFN treatment of A549 cells to the rtAI of each mRNA. (b) Comparison of the log_2_ change in the amount of each human mRNA (including both IRG and non-IRG mRNAs) caused by IFN treatment of A549 cells to the absolute rtAI of each mRNA. Download FIG S3, PDF file, 0.2 MB. Copyright © 2018 Smith et al. 2018 Smith et al. This content is distributed under the terms of the Creative Commons Attribution 4.0 International license.

To determine how the phenotype of reduced inhibition by IFN relates to the other phenotype of modern H3N2 viruses, namely, the preservation of optimal virus replication, we generated two new recombinant viruses with recoded PB1 mRNAs (virus constructs 3 and 4). Using the Udorn PB1 as a starting sequence, constructs 3 and 4 were designed to achieve the same rtAI value as construct 2 by randomly selecting and then iteratively changing only those synonymous codons whose isoaccepting tRNAs differed significantly (adjusted *P* < 0.05) between IFN-treated and untreated cells in the tRNA sequencing results presented in Fig. 3. Constructs 3 and 4 differed in the codons that were randomly selected for changing. The sequences of these two constructs are shown in the accompanying Zenodo citation. Only small numbers of codon changes had to be made, 27 for construct 3 and 25 for construct 4, comprising approximately 2% to 3% of the codons in PB1 mRNA. This percentage range is less than the percentage (approximately 5%) of the codon changes that were introduced into PB1 mRNA to generate the construct 1 and 2 viruses. The latter changes in synonymous codon usage, which were also observed in the PB1 mRNAs of modern H3N2 viruses, include codons that have only a small effect on their rtAI values or which lower the rtAI value.

**FIG 5:**
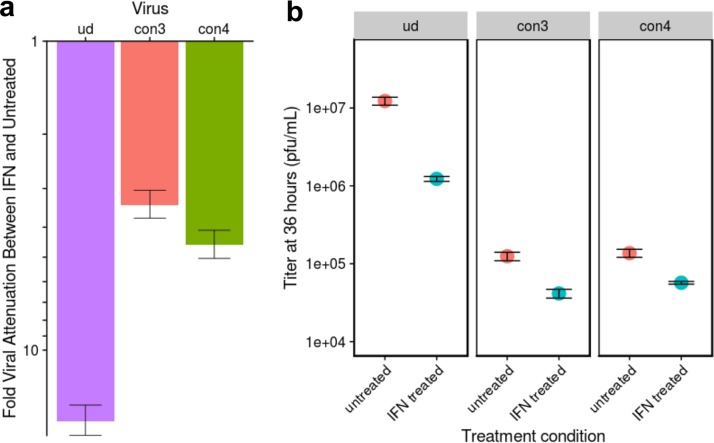
The skewing of PB1 mRNA codons toward the tRNA pools of IFN-treated cells does not explain the other phenotype of modern H3N2 viruses, namely, the retention of optimal virus replication in human cells. (a) Mean fold change in virus titers caused by IFN treatment at 36 h for Ud WT and construct 3 (con3) and construct 4 (con4) viruses. The y axis denotes log values. The error bars denote standard errors. All differences from Ud WT are significant (*P* < 0.05) as determined by two-tailed *t* test. (b) Titers of virus collected in triplicate from the media of cells infected with WT Ud virus and virus constructs 3 and 4 at 36 h postinfection under both the IFN-treated and untreated conditions. Means are reported with error bars denoting standard errors.

As predicted by their rtAI values, the replication of virus constructs 3 and 4 was reduced by IFN treatment 8-fold to 10-fold less than WT Ud virus replication (Fig. 5a), as was the case for virus constructs 1 and 2. However, unlike virus constructs 1 and 2 and modern H3N2 viruses, virus constructs 3 and 4 were attenuated compared to WT Ud virus in both IFN-treated and untreated A549 cells (Fig. 5b), confirming that rtAI values predict only how the PB1 mRNA is translated relatively between IFN-treated and untreated human cells and do not predict overall translation levels or the relative fitness levels of the virus. Importantly, these results show that the tilting of codon usage of PB1 mRNA toward IFN-altered tRNA pools inhibits rather than preserves optimal virus replication, suggesting that the synonymous codon changes that do not contribute to an increase in rtAI values are involved in the retention of optimal replication of influenza A virus in human cells.

### Calu3 cells display a change in synonymous codon usage after IFN treatment consistent with the findings in A549 cells.

**FIG 6:**
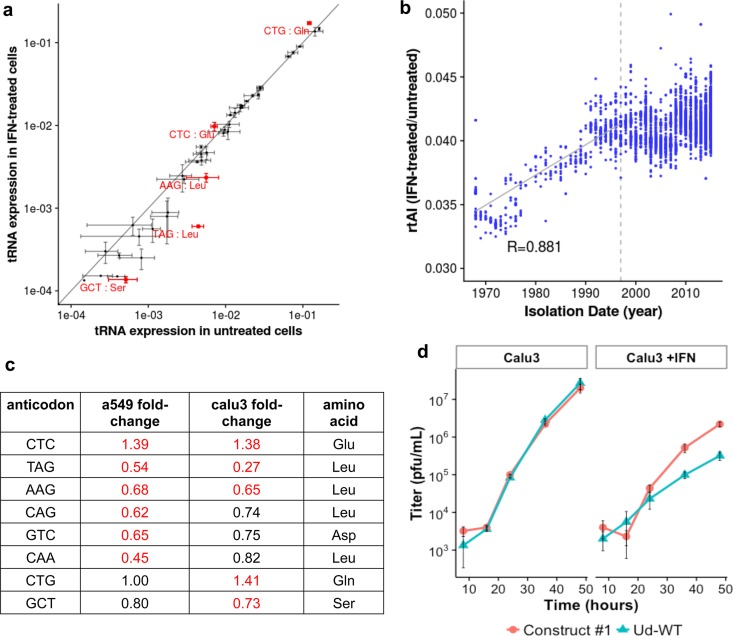
Calu3 cells display a change in synonymous codon usage after IFN treatment consistent with the findings in A549 cells. (a) Proportion of total tRNAs of each tRNA anticodon in untreated cells versus the proportion of total tRNAs in IFN-treated cells. Means are reported with error bars indicating standard errors. Six anticodons (marked in red) show a statistically significant difference between untreated and IFN-treated cells (*P* [adjusted] < 0.05) by DESeq2. (b) The rtAI values, based on quantification of Calu3 tRNA pools, for H3N2 PB1 mRNAs over time. The diagonal solid line represents the linear regression across the first 30 years of circulation, and the 30-year mark is denoted by the dashed vertical line. The *P* value of the linear regression, determined by the reshuffling test, is 0.056. (c) The fold change in tRNA availability due to IFN treatment for all anticodons found to be statistically significant by DESeq in either A549 or Calu3 cells. Red letters indicate that the anticodon was found to be significantly different (*P* adjusted < 0.05) in the indicated cell line. (d) Growth curves, from experiments carried out in triplicate, of construct 1 and WT Ud in Calu3 cells infected at a MOI of 0.01. Mean titers are reported with error bars indicating standard errors.

To ensure that the results observed in A549 cells are not exclusive to this human alveolus-derived cell line, we replicated a subset of our experiments in human bronchial-tube-derived Calu3 cells. Sequencing of tRNAs in IFN-treated and untreated Calu3 cells was carried out as described for A549 cells. Figure 6a shows the plot of the amounts of each tRNA anticodon as a proportion of all tRNAs in untreated cells versus the amounts of the tRNA anticodon as a proportion of all tRNAs in IFN-treated cells. Five tRNA anticodons were found to have shown statistically significant differences (adjusted *P* < 0.05) between the IFN-treated and untreated conditions in Calu3 cells (Fig. 6b). Three of these anticodons, CTC for Glu and TAG and AAG for Leu, were also found in A549 cells, confirming that the levels of tRNAs with these anticodons are increased after IFN treatment of human cells. Two other anticodons identified in the analysis of Calu3 cells were not significantly different (adjusted *P* < 0.05) in A549 cells. While not all tRNA anticodons that passed the adjusted *P* threshold value of <0.05 in A549 cells also passed this threshold in Calu3 cells, and vice versa, the fold change in tRNA availability between IFN-treated and untreated cells was in the same direction for 7 of the 8 anticodons (Fig. 6b). In addition, calculation of the rtAI values for H3N2 PB1 sequences based on the tRNA availabilities in Calu3 cells yielded the same upward trend of rtAI values over the first 30 years of circulation that was observed for A549 cells (Fig. 6c). Another similarity was shown by the fact that the codon-altered construct 1 virus exhibited a significantly lower (approximately 10-fold) reduction in replication resulting from IFN treatment than the WT Ud virus, as was the case in A549 cells (Fig. 6d). We conclude that the overall differences in tRNA availability between the IFN-treated and untreated conditions are similar in Calu3 and A549 cells.

## DISCUSSION

The evolution of the synonymous codons of the avian PB1 gene since its incorporation into the 1968 H3N2 human pandemic virus constitutes a unique experiment in nature that demonstrates how the synonymous codons of an avian influenza A virus gene adapt to the new human host. Unexpectedly, we found that the codon usage of the PB1 mRNA of recent H3N2 viruses enhances replication in IFN-treated human cells approximately 10-fold without affecting replication in untreated cells (Fig. 2), thereby partially alleviating the IFN-induced antiviral state. Here we focus on determining how the codon usage of modern H3N2 viruses reduces the inhibition of virus replication in human cells in which a potent antiviral state has been induced by IFN treatment. We show that reduced IFN inhibition of virus replication is explained by the evolution over time of the synonymous codon usage of PB1 mRNA that is skewed toward the tRNA pools in IFN-treated human cells, which, as shown here, differ significantly from the tRNA pools in untreated human cells. Because the virus phenotype of reduced IFN inhibition correlates with IFN-altered changes in cellular tRNA pools, it is likely that this phenotype results from an increase over time in the translation of PB1 mRNAs in IFN-treated cells. Specifically, such an increase in translation of PB1 mRNAs of modern H3N2 viruses is likely due to their acquisition of a mode of codon usage that better reflects tRNA availabilities in IFN-treated cells. Only a small increase in translation would be expected because only approximately 5% of the PB1 mRNA codons need to be changed to acquire this phenotype. Our results indicate that the change in tRNA availabilities resulting from IFN treatment is predominately an antiviral response and that IFN-regulated (IRG) mRNAs are likely translated similarly in untreated and IFN-treated cells. In fact, host IRG mRNAs appear to specifically avoid codons recognized by isoaccepting tRNAs that change in availability between IFN-treated and untreated cells.

The small increase in the amount of the PB1 protein, which is a subunit of the viral polymerase (17–19), would lead to a small increase in the level of the viral polymerase that catalyzes viral mRNA synthesis and viral RNA replication because the slightly increased PB1 protein level would bring a similar amount of the other polymerase subunits (PB2 and PA) into the viral polymerase complex. It is likely that such a small amount of PB2 and PA would be available in IFN-treated cells because the mRNAs of these two viral polymerase proteins have already adapted to human tRNA pools and hence should be translated more efficiently than PB1 mRNAs that have not yet adapted to human cells. The small increase in the assembled viral polymerase would then result in small increases in the transcription of the three polymerase genes. This process would be repeated during the multiple rounds of polymerase assembly occurring during virus infection, resulting in substantial amplification of the amount of the viral polymerase. This amplification of the viral polymerase would be expected to lead not only to the observed substantial increases in IFN-treated cells of the amounts of PB1-specific RNAs and PB1 protein but also to similar increases in the amounts of all other viral RNAs and viral proteins. In contrast, small changes in the translation of viral mRNAs encoding proteins that are not components of the viral polymerase would not be amplified and hence would not be expected to lead to a measurable phenotype.

Interestingly, the skewing of these PB1 mRNA codons toward the tRNA pools of IFN-treated cells does not explain the other phenotype of modern H3N2 viruses, namely, the retention of optimal virus replication in human cells, as shown by the results presented in Fig. 5. Our results suggest that synonymous codon changes in PB1 mRNA that do not contribute to an increase in rtAI values are involved in the retention of optimal virus replication in human cells. Further studies are needed to determine whether this is the case and, if so, to determine how these codons act. It is likely that the reduction in the inhibition by IFN is constrained by the need to preserve optimal virus replication.

Presumably, one role of the IFN-induced alteration of the tRNA pools of human cells is to reduce the efficiency of translation of viral mRNAs in IFN-treated cells relative to the efficiency in untreated cells and hence is a previously unknown aspect of the antiviral action of IFN. The avian influenza virus-derived PB1 gene of modern H3N2 viruses partially counteracts this IFN antiviral action as a result of the evolution of its synonymous codon usage having skewed toward interferon-altered human tRNA pools. We postulate that such a codon adaptation was also the case for the genes for the other two protein subunits of the viral polymerase (PA and PB2) when they were incorporated into human-infecting influenza A viruses. However, as the introduction of these genes occurred so far in the past, no relevant sequence results are available to enable us to determine whether such a codon adaptation of PA and PB2 occurred. Further, we postulate that enhanced replication in IFN-treated cells plays an important role in the human adaptation of nonhuman virus genes that are incorporated into other human viruses, but only when the nonhuman viral genes encode proteins required for viral RNA or viral DNA synthesis.

Our results show that it took a long time—30 years—for the synonymous codons of PB1 mRNA to adapt to human cells. This long adaptation time likely stemmed from the very small effect of an individual synonymous codon change on virus replication and/or resistance to IFN inhibition, so that synonymous substitutions would be expected to accumulate initially via random genetic drift rather than via selection. However, because multiple synonymous changes of the right kind do eventually confer some adaptive advantage, random genetic drift would then become slightly biased, leading to a trend toward adaptation of synonymous codons occurring over a long time period (decades). In contrast, with nonsynonymous codons, because mutations change an amino acid sequence, a significant effect on virus fitness could result. Consequently, such mutated viruses rapidly outcompete those that do not carry the mutation, so that the adaptation spreads in a short time interval (weeks to months).

The mechanism by which IFN causes the alteration in tRNA pools is not known. This alteration may result from selective transcription of specific tRNA genes, as observed in other systems in which tRNA pool changes occur (27, 28). Also, IFN-induced proteins, for example, the IFIT5 and Schlafen 11 proteins that bind various tRNAs, may play a role (35, 36). In this regard, it was reported more than 40 years ago that extracts from interferon-treated cells inactivated several tRNAs, specifically including Leu tRNAs (37), suggesting that IFN treatment might induce the production of tRNA-specific nucleases.

Recent studies have demonstrated that differences in tRNA pools underlie different states of human cells. One study showed that proliferating human cells and differentiating human cells have different tRNA pools (27). Another study showed that increases in the levels of two tRNAs in human breast cancer cells play an important role in inducing metastatic activity (28). Here we show that the IFN-induced antiviral state is characterized by changes in the amounts of several tRNAs relative to the amounts in untreated cells and that these tRNA changes affect the replication of influenza A virus. Our results, coupled with the recent studies linking changes in the tRNA pools with changes in the state of human cells, highlight the important role of tRNA pools in the regulation of gene expression in human cells.

## MATERIALS AND METHODS

The procedures for growth of A549, Calu3, and MDCK cells and for the generation of Ud viruses encoding codon-altered PB1 mRNAs using plasmid-based reverse genetics have been described previously (38, 39). The codon adaptation index (CAI), as described by Sharp and Li (20), was used to compare the level of codon usage in influenza PB1 mRNAs to human codon usage as described in Text S1 in the supplemental material. Protein levels were measured by immunoblotting, and the levels of viral RNAs in virus-infected cells were determined using quantitative RT-PCR as described in Text S1. High-throughput sequencing of tRNAs using a thermostable group II intron reverse transcriptase (32) was carried out as described in Text S1. The calculations involved in determining the relative tRNA adaptation index (rtAI) and absolute-rtAI are described in Text S1.

### Code availability.

All scripts involved in this study are available in the accompanying Zenodo citation (https://doi.org/10.5281/zenodo.1288883).

### Data availability.

Sequences for codon-altered PB1 constructs are available in the accompanying GitHub repository (https://github.com/wilkelab/influenza_codon_usage), which is also archived on Zenodo (https://doi.org/10.5281/zenodo.1288883). Acknowledgment files for influenza sequences downloaded from the GISAID database are also available in the accompanying GitHub repository and its Zenodo archive.
