# Supplementary material for: Avian Influenza Virus PB1 Gene in H3N2 Viruses Evolved in Humans To Reduce Interferon Inhibition by Skewing Codon Usage toward Interferon-Altered tRNA Pools
Source: mBio. 2018 Jul 3;9(4):e01222-18. doi: 10.1128/mBio.01222-18 (PMC6030557; doi:10.1128/mBio.01222-18)
Supplement: FIG S1 [file mbo004183961sf1.pdf]

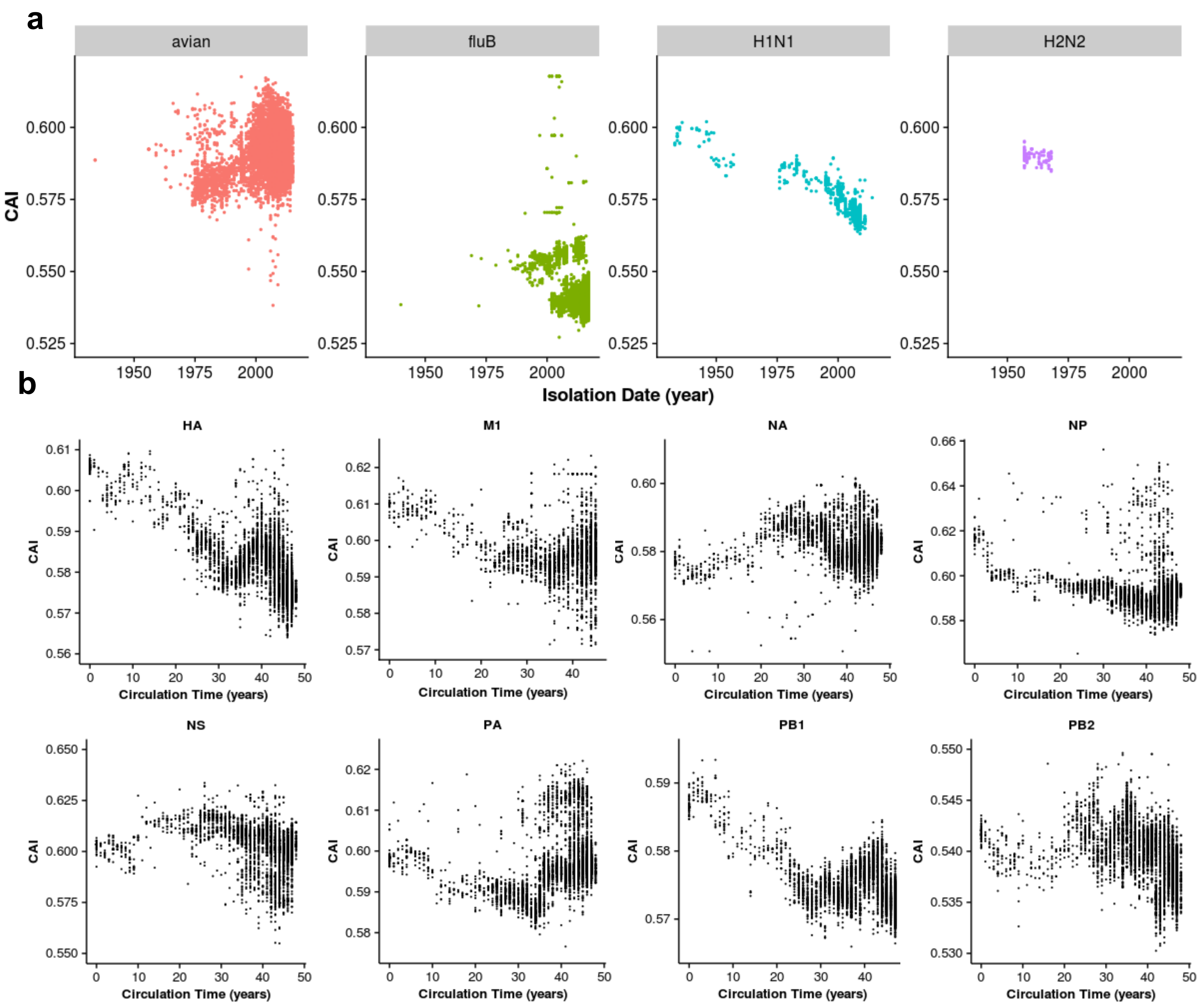


**Figure S1.** The CAI values over time since 1968 for: **a**, avian virus, influenza B virus, H1N1, and H2N2 PB1 genes; and **b.** each segment of H3N2 viruses
