# Supplementary material for: Avian Influenza Virus PB1 Gene in H3N2 Viruses Evolved in Humans To Reduce Interferon Inhibition by Skewing Codon Usage toward Interferon-Altered tRNA Pools
Source: mBio. 2018 Jul 3;9(4):e01222-18. doi: 10.1128/mBio.01222-18 (PMC6030557; doi:10.1128/mBio.01222-18)
Supplement: TEXT S1 [file mbo004183961s1.pdf]

Avian influenza PB1 gene in H3N2 viruses evolved in humans to reduce interferon inhibition by skewing codon usage toward interferon-altered tRNA pools

Supplemental Materials and Methods

The Codon Adaptation Index (CAI)

The Codon Adaptation Index (CAI), as described by Sharp and Li (1), was used to compare codon usage in influenza PB1 mRNAs to human codon usage. The 9,000 full-length sequences of H1N1, H2N2 and , and H3N2 PB1 segments of influenza A virus used in this research came from GISAID (http://gisaid.org). Acknowledgements for sequences used in this study are available in Supplemental Material File 1. The reference set of human genes was comprised of the most highly expressed genes across at least five tissues, as determined from the Affymetrix Gene Chip Human Genome U95Av2, which was downloaded from the EMBL-EBI Expression ATLAS database. The sequences of the 200 most highly expressed human genes across more than five tissues were downloaded from the NCBI Entrez database. These genes were used to calculate the RSCU of each codon. The CAI for each H3N2 PB1 mRNA was calculated and plotted against the circulation time, the number of years the PB1 segment has been circulating in the human population since its introduction in 1968 into human-infecting H3N2 viruses. To demonstrate that the resulting trend in CAI values is significant, and not caused by the phylogenetic relationship between influenza A virus isolates, a reshuffling test was performed whereby the RSCU for each possible codon was switched to that of a randomly selected synonymous codon. This reshuffling was done at each position in the alignment of PB1 genes. The CAI for each PB1 gene was

then calculated based on these reshuffled RSCU values, and the correlation coefficient between reshuffled-CAI and year of isolation for the isolates was determined for the first 30 years of virus circulation in humans. The reshuffling test was performed 1,000 times and the p-value was determined by the proportion of reshuffled tests that produced a correlation coefficient with an absolute value higher than that which was found in the original data.

Cells and viruses.

A549, Calu3 and MDCK cells were grown in Dulbecco's modified Eagle medium (DMEM) supplemented with 10% heat-inactivated fetal bovine serum. Wild-type (WT) Ud virus and Ud viruses encoding codon-altered PB1 mRNAs were generated by plasmid-based reverse genetics as described previously (2, 3). All eight genomic RNA segments of recombinant viruses were sequenced. Virus stocks were grown in 10-day-old fertilized eggs, and virus titers were determined by plaque assays in MDCK cells. For multiple-cycle virus growth, A549 or Calu3 cells were infected with virus at a multiplicity of infection (moi) of 0.001 PFU/cell. After 1 hour of adsorption at 37°C, the cells were washed once with phosphate-buffered saline (PBS), replenished with medium containing 2.5 μg/ml N-acetylated trypsin (NAT), and incubated at 37°C. For single-cycle growth, A549 cells were infected with virus at a moi of 5 PFU/cell. Growth curves were carried out in triplicate, with three independent cultures of cells being infected with virus.

Codon altered Ud PB1 mRNAs

Constructs #3 and #4 were designed to achieve the same rtAI value of construct #2 by iteratively changing only those synonymous codons whose isoaccepting tRNAs differed significantly (ρ-adjusted<0.05) in the tRNA sequencing results between IFN-treated and untreated cells. The codons to change were determined by a hillclimbing algorithm available in the codon_tools python package (https://github.com/clauswilke/codon_tools).

Immunoblots

Infected cell experiments: A549 cells treated or not treated with IFN (1000U/ml) (Pestka Biological Laboratories, Piscataway NJ) for 36 hours were infected with WT Ud virus or a codon-altered Ud virus (constructs #1-#4) at a moi of 1 pfu/cell. Cells were collected 9 hours after infection, and cell extracts were analyzed with immunoblots probed with the indicated antibodies. Protein bands were quantified using ImageJ (4), with actin bands used as a loading control. The antibody against the NS1 protein was characterized previously (5). Rabbit Ab against PB1 was provided by Krister Melen and Ikka Julkunen (Finnish National Institute for Health and Welfare), and then validated (5). Monoclonal Abs against PA and PB2 were provided by Juan Ortin (6). The polyclonal goat Ab against HA, NP and M1 was provided by Robert A. Lamb (7).

qPCR measurement of viral RNAs in infected cells

A549 cells treated with IFN (1,000U/ml) or mock treated for 36 hours were infected with WT Ud virus or construct #1 or construct #2 virus at a moi of 1 pfu/cell. Three hours

after infection total RNA was extracted with trizol (Invitrogen). An aliquot of each RNA sample was reverse transcribed with oligo dT, followed by amplification with the segment-specific forward and reverse primers listed below to measure mRNA levels by the Vii7 real-time PCR system using Power SYBR Green PCR Master Mix (Thermo Fisher Scientific). An equal aliquot of each RNA sample was reverse transcribed using the segment-specific RT primers listed below, followed by the same amplification described above to measure vRNA levels by real-time PCR, which was carried out in triplicate. The mRNA and vRNA levels were normalized with the real-time PCR measurements of β-actin mRNA levels. The final fold inhibition fold calculated with ΔΔCT methods.

Primers used:

actin reverse primer: GGTCTCAAACATGATCTGGG

actin forward primer: GCGACGAGGCCCAGAGCAAG

PB1 RT primer for vRNA: GAATGGATGTCAACCCGAC

PB1 forward Primer: CGAGGAGATCATTCGAGCTAAAG

PB1 reverse Primer: TTAAGCAGACTTCAGGGATGTG

NP vRNA RT primer: GTCCCAAGGCACCAAACGGTCTTATG

NP Forward: GAACTTCTGGAGAGGTGAGAATG

NP Reverse: GCTCTTTGTGCAGCTGTTTG

M vRNA RT primer: GAAAGATGAGCCTTCTGACCG

M Forward: CAAATGGTGCAGGCAATGAG

M Reverse: CAAGAGGGTCACTTGAATCGT

High-throughput sequencing of tRNAs from IFN-treated and untreated A549 and Calu3 human cells

Two 15cm tissue culture plates of either A549 or Calu3 cells were treated with IFN to a final concentration 1,000U/mL or with an equal volume of PBS. After 36 hours, total RNA was extracted with trizol, and tRNA was purified using urea-PAGE. The gel was stained with ethidium bromide and the band containing tRNA was excised from the gel. Three samples of each tRNA population were used. tRNA was eluted from the polyacrylamide gel slice by electroelution with D-Tube Dialyzer Midi, MWCO 6-8 kDa (Novagen), and then concentrated by ethanol precipitation. tRNAs were deacylated by incubation in 100ul of 0.1M Tris-HCl (pH 9.0) for 45 minutes at 37°C. Deacetylated tRNAs were concentrated by ethanol precipitation. Libraries were made using the TGIRT template switching reactions as previously described (8). The purity of the libraries was verified using a high sensitivity bioanalyzer. Sequencing was performed by the University of Texas Genomic Sequencing and Analysis Facility on either Illumina HiSeq 4000 or illumina MiSeq machines. Sequencing reads were aligned to the GRCh38 human reference genome with bowtie2, using the default settings and reporting only the best match. Using SAMtools and BEDtools, the intersection of the human reference alignments with a reference set of tRNA genes for GRCh38 (kindly provided by the Alan Lambowitz) was determined, and the read counts for each tRNA gene were determined. Counts for each gene were pooled by anticodon. Differential expression of anticodons was analyzed with the R package DEseq2.

The relative tRNA Adaptation Index (rtAI) and the absolute-rtAI

The first step to calculating rtAI involves finding the sum of all isoaccepting tRNAs for each codon in the IFN treated and untreated conditions. The isoaccepting tRNAs (isotRNA) for each codon in each treatment are determined using a table of codon-anticodon pairings:

$$isotRNA_{i\ IFN} = \sum_{j=1}^{t_i} x_{ij\ IFN-treated}$$

$$isotRNA_{i\ untreated} = \sum_{j=1}^{t_i} x_{ij\ untreated}$$

where x_{ij} is the number of tRNA reads mapped for the *j*th tRNA capable of recognizing the *i*th codon for the given treatment condition (either IFN-treated or untreated), and t_i is the number of alternative tRNAs that can recognize the *i*th codon. The relative synonymous codon usage by tRNA (RSCUt) of each codon in each treatment is then calculated as follows:

$$RSCUt_{ih\ IFN-treated} = \frac{isotRNA_{ih\ IFN-treated}}{\sum_{h=1}^{n_h} isotRNA_{ih\ IFN-treated}}$$

$$RSCUt_{ih\ untreated} = \frac{isotRNA_{ih\ untreated}}{\sum_{h=1}^{n_h} isotRNA_{ih\ untreated}}$$

where $isotRNA_{ih}$ the number of isoaccepting tRNAs for the ith codon for the *h*th amino acid for the given treatment, and n_h is the number of alternative codons for the *h*th amino acid. The rtAI is then calculated as the mean of the log ratio of the IFN-treated RSCUt over the untreated RSCUt for each codon across all codons in a gene:

$$rtAI = \frac{\sum_{k=1}^{L} log \frac{RSCUt_{k\ IFN-treated}}{RSCUt_{k\ untreated}}}{L}$$

where $log\frac{RSCUt_{k\,IFN-treated}}{RSCUt_{k\,untreated}}$ is the log ratio of the RSCUt values for the *k*th codon in the gene and *L* is the number of codons in the gene. In our tRNA sequencing experiment, there was a single tRNA that was greatly underrepresented, with an average of less than 100 reads in each replicate and treatment condition. As we could not expect accurate quantification with such a low read number, it was excluded from our analysis. This exclusion only has a significant effect on one codon, TTA, for which the underrepresented tRNA was the only isoaccepting tRNA, and so this codon was ignored in the calculation of rtAI. The rtAI was calculated for the GISAID dataset described above. The upwards trend observed in the first 30 years of circulation has a correlation coefficient of 0.807. To show that this trend is significant, and not caused by the phylogenetic relationship between influenza virus isolates, a reshuffling test was performed as described above. The correlation coefficient (R) of the non-reshuffled data was significantly higher than the correlation coefficients *R* of the reshuffled RSCU (p = 0.043).

The calculation of absolute-rtAI is very similar to the calculation for rtAI. The only difference is in the upper term that is being summed in the final equation for rtAI. Instead of the log ratio of the RSCUt values being averaged across the gene, the absolute value of the log ratio is averaged, so the final equation in the determination of absolute-rtAI is as follows:

$$rtAI = \frac{\sum_{k=1}^{L} |log\frac{RSCUt_{k\,IFN-treated}}{RSCUt_{k\,untreated}}|}{L}$$

References

1. **Sharp PM, Li WH.** 1987. The codon Adaptation Index--a measure of directional synonymous codon usage bias, and its potential applications. Nucleic Acids Res **15:**1281-1295.
2. **Takeda MP, A.; Shuck, K.,;Pinto, L. H.; and Lamb, R. A.** 2002. Influenza a virus M2 ion channel activity is essential for efficient replication in tissue culture. J Virol **76:**1391-1399.
3. **Twu KY, Noah DL, Rao P, Kuo RL, and, Krug RM.** 2006. The CPSF30 binding site on the NS1A protein of influenza A virus is a potential antiviral target. J. Virol. **80:**3957-3965.
4. **Abramoff MD, Magalhaes PJ, Ram SJ.** 2004. Image processing with ImageJ. Biophotonics International **11:**36-43.
5. **Kuo RL, Zhao C, Malur M, Krug RM.** 2010. Influenza A virus strains that circulate in humans differ in the ability of their NS1 proteins to block the activation of IRF3 and interferon-beta transcription. Virology **408:**146-158.
6. **Ochoa M, Barcena J, de la Luna S, Melero JA, Douglas AR, Nieto A, Ortin J, Skehel JJ, Portela A.** 1995. Epitope mapping of cross-reactive monoclonal antibodies specific for the influenza A virus PA and PB2 polypeptides. Virus Res **37:**305-315.
7. **Chen BJ, Leser GP, Morita E, Lamb RA.** 2007. Influenza virus hemagglutinin and neuraminidase, but not the matrix protein, are required for assembly and budding of plasmid-derived virus-like particles. J Virol **81:**7111-7123.

8. **Mohr S, Ghanem E, Smith W, Sheeter D, Qin Y, King O, Polioudakis D, Iyer VR, Hunicke-Smith S, Swamy S, Kuersten S, Lambowitz AM.** 2013. Thermostable group II intron reverse transcriptase fusion proteins and their use in cDNA synthesis and next-generation RNA sequencing. RNA **19:**958-970.
