# Supplementary material for: Avian Influenza Virus PB1 Gene in H3N2 Viruses Evolved in Humans To Reduce Interferon Inhibition by Skewing Codon Usage toward Interferon-Altered tRNA Pools
Source: mBio. 2018 Jul 3;9(4):e01222-18. doi: 10.1128/mBio.01222-18 (PMC6030557; doi:10.1128/mBio.01222-18)
Supplement: FIG S3 [file mbo004183961sf3.pdf]

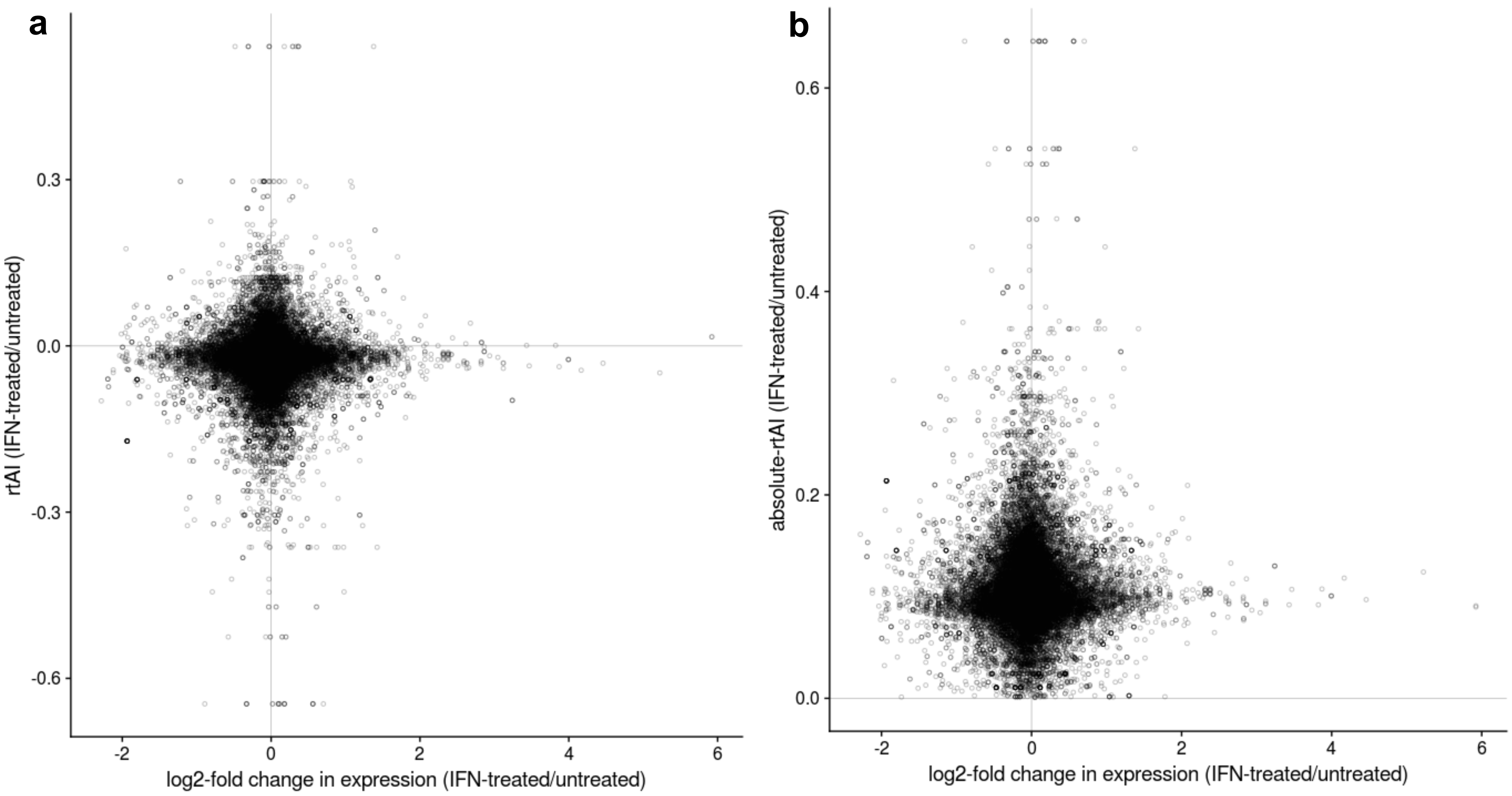


**Figure S3. a**, Comparison of the log2 change in the amount of each human mRNA (both IRG and non-IRG mRNAs) caused by IFN treatment of A549 cells to the rtAI of each mRNA. **b.** Comparison of the log2 change in the amount of each human mRNA (both IRG and non-IRG mRNAs) caused by IFN treatment of A549 cells to the absolute-rtAI of each mRNA.
